# Percutaneous Nephrostomy in Complicated Urinary Tract Infections

**DOI:** 10.7759/cureus.26682

**Published:** 2022-07-09

**Authors:** Utsav Mondal, Stalin Viswanathan, Sreerag Sreenivasan Kodakkattil

**Affiliations:** 1 General Medicine, Jawaharlal Institute of Postgraduate Medical Education and Research, Pondicherry, IND; 2 Urology and Renal Transplantation, Jawaharlal Institute of Postgraduate Medical Education and Research, Pondicherry, IND

**Keywords:** pyonephrosis, pyelonephritis, obstructed urinary system, complicated urinary tract infection, percutaneous nephrostomy

## Abstract

Background

The study aimed to determine the various indications for percutaneous nephrostomy (PCN) primarily in patients with a urinary tract infection and to determine the various complications arising in these patients due to the procedure.

Materials and methods

A retrospective study of five-year data based on registers of the Department of Urology was performed. Among 716 patients, 226 inpatient data were obtained, curated, and analyzed. Indications for PCN, the periprocedural complications, the PCN's duration, details of antibiotics, risk factors for UTI, development of acute kidney injury, and renal replacement therapy were recorded.

Results

Patients were mostly female (53.1%, n=120/226). Malignancy (n=109, carcinoma cervix=68/109) and nephrolithiasis (n=70) contributed to 79.2%. Infections such as pyelonephritis, renal abscess, perinephric abscess, and genitourinary tuberculosis were identified in 47. Infectious diseases were significantly associated with younger age, female gender, diabetes, and prior pyelonephritis. PCN was placed at a median of two days after admission, and bilateral PCN was placed in 36 (15.2%) patients. Hydroureteronephrosis (probably infected) was an indication for PCN in 164/226 patients. In 33 patients with an infectious disease, PCN was performed for an obstructed urinary system. One major and two minor complications gave a rate of 0.06% for patients with infections, which was less than the accepted threshold of 4%.

Conclusions

We intended to study the utility and problems with placing a PCN catheter in patients with complicated urinary infections. We conclude that PCN is a safe and effective procedure in urinary tract infections with obstructed drainage.

## Introduction

Percutaneous nephrostomy (PCN) is a minimally invasive procedure performed for upper urinary tract drainage [1,2] Adjunctively, it is used for lithotripsy, ureteral stents, and urinary diversion [2]. Complicated upper urinary tract infection (UTI) is defined by the presence of risk factors such as diabetes and anatomical abnormalities [3] About 0.1% to 10% of those undergoing PCN develop some minor or major complications [2]. In patients with pyonephrosis, the septic complications may reach 25%. About 90% of all procedures for urinary drainage are performed for obstruction of the urinary tract arising due to stones or malignancy [4]. Data regarding pyelonephritis and infected hydronephrosis are scarce. Complications following PCN may increase morbidity and mortality; new septic complications may often be difficult to attribute to the procedure [4]. The frequency of complications related to PCN for urinary infection has been occasionally studied [5]. Hence, we decided to attempt a five-year audit of PCN procedures that were necessitated for complicated UTIs such as pyelonephritis. The manuscript is available as a preprint at https://doi.org/10.21203/rs.3.rs-1515128/v1.

## Materials and methods

The study aimed to determine the various indications for PCN primarily in patients with a urinary tract infection and to determine the various complications arising in these patients due to the procedure. The study was performed as an ICMR-STS project (2020-01574). The Institute Ethics Committee approved the study (JIP/IEC/2020/237). The registers of the Urology Department were accessed for patients who underwent a PCN between January 1, 2015 and December 31, 2019. A list of these patients was provided to the Medical Records Department for obtaining the case records. Demographics, symptoms, signs, and investigations were noted. Indications for PCN, the periprocedural complications, duration that the PCN remained in the patient, and details of antibiotics, risk factors for UTI, development of acute kidney injury, and renal replacement therapy were recorded.

Inclusion criteria included all patients ≥18 years undergoing a PCN during the study period. We excluded patients who had PCN placed on an outpatient basis. Patients admitted to the Department of Medicine for complicated urinary infections which required PCN were focused upon. Data were collected using the Epicollect app v.5 and analyzed using IBM SPSS for Windows version 22. Frequencies of discrete variables such as risk factors, type of UTI, antibiotics, organisms, indications, and periprocedural complications of PCN were calculated and analyzed using the Chi-square test or Fischer’s exact test. Means ± SD was calculated for continuous variables such as age, duration of illness, duration of hospitalization, duration of PCN, and laboratory variables. Patients with infectious diseases were compared with noninfective conditions like malignancy and nephrolithiasis. A p-value of ≤0.05 was considered statistically significant. The data set is available at https://doi.org/10.6084/m9.figshare.19459733.

## Results

**Figure 1:**
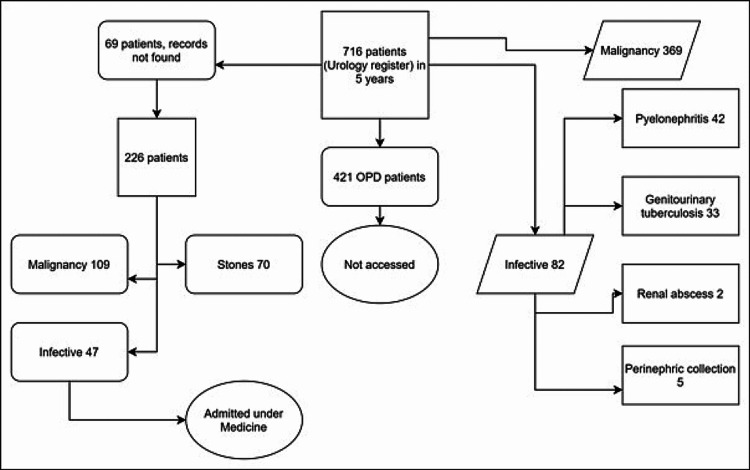
Study flow diagram

**Figure 2:**
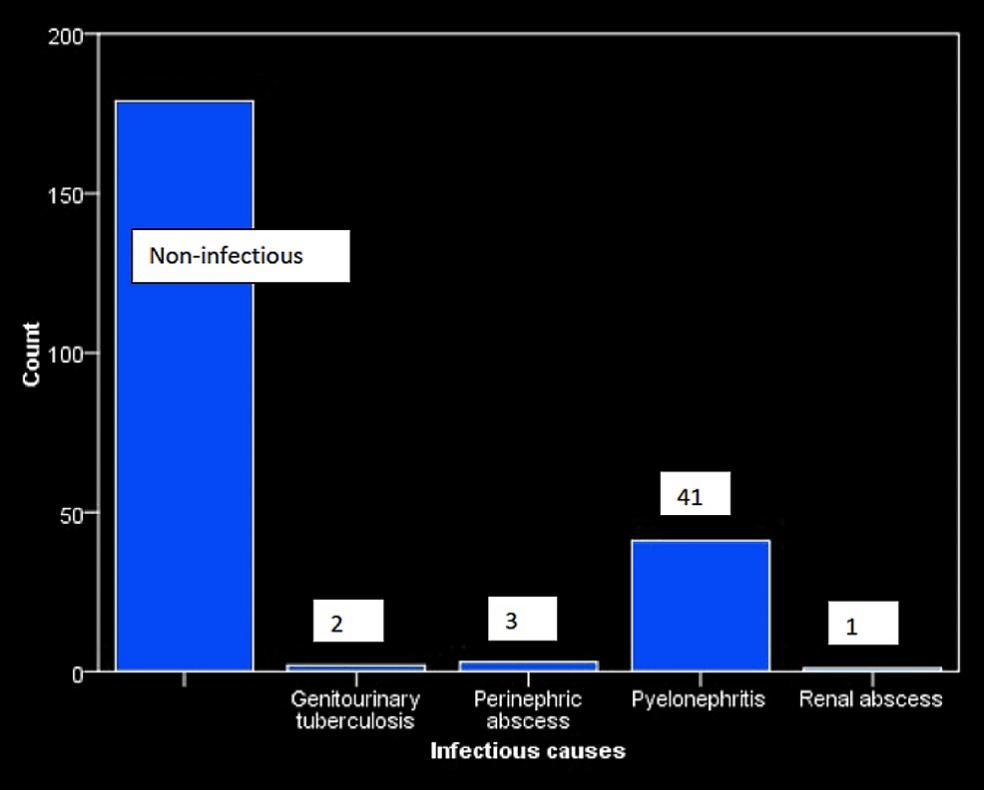
Infectious etiologies in patients requiring percutaneous nephrostomy

There were 226 patients in our study with 120 (53.1%) females (Figure 1), with a mean age of 50.88±14.03 years (range, 5 to 86 years). Forty seven were admitted to the Department of Medicine for urinary tract infections (Figure 2).

**Figure 3:**
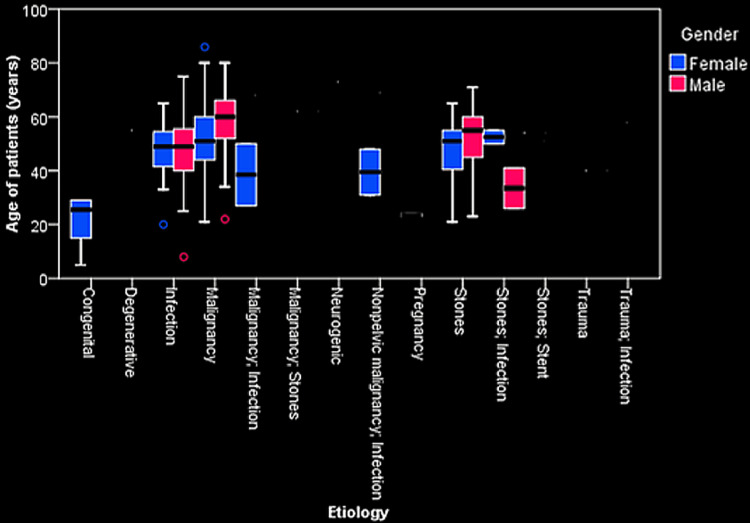
Boxplot shows the relationship between the various etiologies, gender, and age

The majority were being treated in Urology 145 (64.2%), while lesser numbers were under the Departments of Gynecology 14 (6.2%) and Radiation Oncology 20 (8.8%). Malignancy (n=109) and nephrolithiasis (n=70) contributed to 79.2% of the total study population (Figure 3).

Carcinoma cervix (n=68) and carcinoma bladder (n=20) were the most common malignant disorders. Non-pelvic malignancies involving the breast, esophagus, and gall bladder were also seen, but patients with these malignancies had been admitted for acute pyelonephritis. Chronic kidney disease (CKD) was the most common comorbid illness, seen in 59 patients (26.1%), while diabetes and hypertension were observed in 16.8% (n=38) and 16.4% (n=37), respectively.

Infectious diseases were significantly associated with younger age, female gender, diabetes, and prior pyelonephritis (Table 1). Fever, higher pulse rate, and tachypnea were also significantly associated with infections (Table 1). Redo PCN and attempts were significantly related to non-infectious disorders. Cefoperazone-sulbactam was used in nearly half the patients (n=95), while 49 (21.7%) received two or more antibiotics. PCN was placed a median of two days after admission, and bilateral PCN was placed in 36 (15.2%) patients. Hydroureteronephrosis (probably infected) was an indication for PCN in 164/226 patients. In 42/226 patients, ≥ 2 attempts at placing PCN were made, and a redo PCN was performed in 31 patients. In 34 patients with an infectious disease, PCN was performed for an obstructed urinary system (Table 2).

**Table 1:** Baseline information, laboratory investigations, and treatment in infectious and non-infectious disorders HUN-hydroureteronephroses, PCN-percutaneous nephrostomy

Variable	Infectious	Non-infectious	Significance	Variable	Infectious	Non-infectious	Significance
Age of patients (years)	47.87±14.49	51.66±13.84	0.09	Urea (mg/dL)	90.61±55.02	108.54±234.01	0.64
Gender Female (n)	18	102	0.02	Creatinine (mg/dL)	4.27±3.05	16.53±135.53	0.58
Males (n)	29	77		Sodium (mEq/L)	130.79±8.17	131.79±12.99	0.67
Duration of stay (days)	14.94±15.01	11.82±11.69	0.12	Potassium (mEq/L)	4.40±0.81	4.90±3.25	0.37
Malignancy (n)	8	101	<0.001	pH	7.27	6.95	0.82
Prior malignancy (n)	3	35	0.03	Aspartate transaminase (IU/L)	33.41±19.30	27.66±16.57	0.22
Nephrolithiasis (n)	5	65	0.001	Alanine transaminase (IU/L)	24.00±18.98	29.28±80.38	0.78
Prior pyelonephritis (n)	8	13	0.04	Alkaline phosphatase (IU/L)	169.15±105.02	193.01±169.78	0.63
Diabetes mellitus (n)	12	26	0.07	Total protein (g/dL)	5.93±1.10	6.52±1.38	0.16
Hypertension (n)	10	27	0.37	Serum albumin (g/dL)	2.68±0.77	3.84±4.77	0.38
Chronic Kidney Disease (n)	17	42	0.11	Unilateral HUN (n)	17	64	0.95
Alcohol (n)	7	19	0.41	Bilateral HUN (n)	20	81	
Smoking (n)	3	16	0.57	Antibiotics ≥2	12	37	0.47
Oligoanuria (n)	5	37	0.11	Other cephalosporins*	9	12	0.009
Fever (n)	16	24	0.001	Cefoperazone- sulbactam	16	79	0.21
Hematuria (n)	1	15	0.13	Amikacin	10	24	0.17
Lower urinary tract symptoms (n)	5	23	0.68	Quinolones	14	50	0.80
Fluid overload (n)	7	17	0.28	Piperacillin- tazobactam	2	6	0.78
Flank pain (n)	12	47	0.92	Right-sided PCN(n)	22	64	0.16
Colicky pain (n)	1	9	0.39	Left-sided PCN (n)	12	62	0.06
Uremic symptoms (n)	4	29	0.18	Bilateral PCN (n)	7	29	0.82
Pulse rate (beats/min)	93.07±12.86	87.22±13.13	0.01	Attempts≥2 (n)	3	39	0.01
Systolic blood pressure (mmHg)	120.37±19.99	123.78±24.99	0.42	Redo PCN (n)	45	150	0.03
Respiratory rate (breaths/min)	23.81±15.69	20.24±7.62	0.09	Postop pain>48h (n)	2	22	0.11
Pallor (n)	12	48	0.84	Other complications (n)	3	17	0.50
Edema (n)	4	15	0.97	Complications (n)	5	42	0.13
Renal angle fullness (n)	8	29	0.73	Intensive care stay (n)	2	11	0.62

**Table 2:** Indications for PCN in infectious and non-infectious diseases HUN-hydroureteronephrosis

	Non-infectious	Genitourinary tuberculosis	Perinephric abscess	Pyelonephritis	Renal abscess	Total
Indication not clear	27	1	-	5	-	33
HUN/pyonephrosis	105	-	2	26	1	134
HUN/pyonephrosis, Nephrolithiasis	24	-	-	3	-	27
HUN/pyonephrosis, Perinephric collection	-	-	-	1	-	1
HUN/pyonephrosis, Pyelonephritis emphysematous	-	-	-	1	-	1
HUN/pyonephrosis, Ureteric stenosis	1	-	-	-	-	1
Nephrolithiasis	18	-	-	1	-	19
Perinephric collection	-	-	1	-	-	1
Pyelonephritis emphysematous	-	-	-	4	-	4
Renal abscess	-	1	-	-	-	1
Ureteric stenosis	3	-	-	-	-	3
Vesico-vaginal fistula	1	-	-	-	-	1

Complications were reported in 8.8% (20/226); pain persisting for ≥48 hours and requiring tramadol or narcotics was observed in 24 (10.6%). Secondary displacement, unusual location or hematuria were not reported. Altered sensorium, dyspnea, and vomiting could not be clearly attributed to the procedure or pre-existing illness. Only three significant complications-fecaluria and slippage of the catheter were noticed among patients with infectious diseases at the rate of 0.06%, 0.02%, and 0.04%, for total, major, and minor complications, respectively (Table 3).

**Table 3:** Complications of PCN in infectious and non-infectious diseases

	Non-infectious	GUTB	Perinephric abscess	Pyelonephritis	Renal abscess	Total
Blockage of tube	2	-	-	-	-	2
Dyspnea, Altered Sensorium	1	-	-	-	-	1
Failure	1	-	-	-	-	1
Faecaluria	-	-	-	1	-	1
Hemorrhage	1	-	-	-	-	1
Pain	17	-	-	-	-	19
Pain, Altered Sensorium, Vomiting	1	-	-	-	-	1
Pain, Blockage of tube	1	-	-	-	-	1
Pus Discharge	1	-	-	-	-	1
Slippage of catheter	7	-	1	1	-	9
Slippage of catheter; pain	1	-	-	-	-	1
Urine leak	3	-	-	-	-	3

Overall, complications were significantly associated with >1 attempt (p=<0.001) at PCN placement and ICU stay (p=0.001). *Escherichia coli *constituted 17 positive cultures, while other organisms such as *Klebsiella pneumoniae* 5, Enterococcus 4, Acetobacter 1, *Pseudomonas aeruginosa* 4, methicillin-resistant *Staphylococcus aureus* 2, methicillin-sensitive *S. aureus* 1, and *Candida albicans *2 contributed to the urinary tract infection of these patients (36/47).

## Discussion

The PCN catheter was used for the first time in 1954 to decompress hydronephrosis [4]. Goodwin et al. first published the utility of PCN in their 15 patients with HUN [6]. Following the USG-guided placement of PCN in 1974, the success rates have increased over the years from the initial 75%. Complicated urinary tract infections are those that generally require intravenous antibiotics due to the risk of urosepsis in patients with anatomical or other predisposing factors such as diabetes, pregnancy, nephrolithiasis, and malignancy [3]. Emergency PCN may be required in upper urinary tract infections to prevent sepsis [4]. Contraindications to the procedure would include severe hyperkalemia, uncorrected coagulopathy, and uncontrolled hypertension, all of which are seen in patients admitted to the medical departments [7].

Females are generally more prone to urosepsis [8], but it was more common among males in our study (29 vs 18), probably due to more diabetes (23 vs 15, p-value=0.06), CKD (37 vs 22, p-value=0.007), nephrolithiasis (49 vs 21, p-value<0.001) and prior pyelonephritis (12 vs 9, p-value=0.32); interestingly, malignancies were commoner in females (30 vs 79, p-value<0.001), but malignancies coexisting with pyelonephritis were seen only in eight (n=101) patients with a p-values of <0.001. PCN is the first-line therapy in infected hydronephrosis and an early adjunctive therapy in patients with emphysematous pyelonephritis [5]. Thirty-three (33/47) of our patients with infection had an obstructed system that needed PCN.

PCN is part of the initial treatment in patients with emphysematous pyelonephritis [9]. The PCN was used in most of our patients to relieve obstruction (renal calculi, pregnancy-related hydronephrosis, and pyonephrosis) and obtain access to the renal system (removal of forgotten stents and percutaneous nephrolithotomy) [4]. Only three of our patients underwent the procedure for urinary diversion; none had diagnostic testing through the PCN [10]. Relief of urinary obstruction was the primary cause for placement of PCN; this was the case even in malignancy, where 65.2% of all malignancies needed PCN due to HUN. Overall, 82.5% of the total (n=226) had an obstructed system necessitating PCN, which was similar to world literature [10].

Complications arising out of PCNL and PCN range from 0.05 to 3% to 2%-10%, respectively [10,11]. Significant complications are seen in less than 1% [5]. Severe complications generally include new sepsis, hemorrhage needing transfusion, and trauma to adjacent structures. Major complications of PCN that have been described in the literature include organ or viscus puncture, hemorrhage requiring transfusion, and sepsis; minor complications include tube blockage requiring further intervention, minor hemorrhage, and urinary extravasation [12].

The rate of complications (0.06% in the infectious group) was lower than the threshold of 4%, according to the Society of Interventional Radiology Standards of Practice Committee [2]. Displacement of catheters can range up to 30% over many months and low as 1% in the early postoperative period [10]. There was a 1.4% incidence of slippage of catheters in our study (n=10). Bacteriuria is a risk factor for fever following PCN, possibly present in most cases, but none reported postoperative fever. Colonic perforation has been reported in left-sided procedures, elderly patients, and those with a distended colon [13] The lone patient with faecaluria had had bilateral PCN placement done.

In the study by Watson et al., 315 patients who underwent PCN, 187 (49.8%) had a calculus for the cause of obstruction and 37 without a determined obstructive cause [5]. Wah et al. studied 218 PCN placements- half of them were due to malignancy and only 4% were due to renal stones [12]. The proportion of pyelonephritis and infective conditions was not known. Contrastingly, stones constituted 31% of our study, while infections contributed to 20.8%. Minor complications were 11% [12].

Successful PCN was placed in the first attempt in 214/218 patients using the Seldinger technique in the study by Watson et al., while 81% (n=183) was achieved in the first attempt in our study [5]. Overall, only 2.8% developed complications, both major and minor [5]. The only major complication in our study was faecaluria, which was conservatively managed. A 10-year Turkish study of 354 patients showed a major and minor complication rate of 11% and 7.7%, respectively [6]. They had 66 patients with pyonephroses, but whether they were initially pyelonephritis, to begin with, is not clear [6].

Previously, a one-and-a-half-year prospective audit of PCN from our institution studied 368 PCN placements (344 patients) with infective conditions in 40 patients. Success rate and complications were studied [14]. A 4.2% major and 27.4% minor complication rate was found. A higher minor complication rate was attributed to a longer follow-up period of one month and the inclusion of urinary tract infection and dislodgement [14]. Ours was retrospective with a focus only on inpatients. We studied 262 PCN placements in 226 patients. Thirty one of these were redo PCNs for complications such as blockage, urine leak, and slippage of the PCN catheter.

Limitations

The modified Clavien classification system of grading complications in PCN was not used. We had included persistent pain≥ 48hours and requiring tramadol, or higher as minor complications. We have not been able to fully explore the stress and discomfort to the patient caused by the PCN. Death and discharge data were not available for many of our patients, and hence these could not be used in the final analysis. Patients with either malignancy or nephrolithiasis and coexisting infection could not be ascribed to postoperative or preoperative causes. The duration of nephrostomy drainage was not studied. We could not study all the confirmed cases of pyelonephritis admitted during the same period under the Department of Medicine, which would have given a better idea of the proportion of patients with infection requiring a PCN. The grading of hydronephroses and hence, the severity and duration of obstruction could not be clarified. The total number of patients who underwent renal replacement therapy could not be accurately determined due to missing data in the case records. Urine culture reports were not available in all patients. Higher BMI has been shown to be a poor prognostic indicator in conditions such as emphysematous pyelonephritis. Due to the retrospective nature of the study, HbA1c and BMI were not available for many patients.

## Conclusions

PCN is a relatively safe procedure used in the setting of malignancies, nephrolithiasis, and infections such as pyelonephritis. Infective patients were younger and less likely female when compared to patients with other disorders. The post-procedural complications in medical patients (presenting with pyelonephritis or renal abscess) were very few and minor.
